# Computerized surveillance of opioid-related adverse drug events in perioperative care: a cross-sectional study

**DOI:** 10.1186/1754-9493-3-18

**Published:** 2009-08-11

**Authors:** Julie A Eckstrand, Ashraf S Habib, Abbie Williamson, Monica M Horvath, Katherine G Gattis, Heidi Cozart, Jeffrey Ferranti

**Affiliations:** 1 Duke Health Technology Solutions, Duke University Health System, Durham, North Carolina, USA; 2 Department of Anesthesiology, Duke University School of Medicine, Durham, North Carolina, USA; 3 Department of Pharmacy, Duke University Health System, Durham, North Carolina, USA; 4 Division of Neonatology, Department of Pediatrics, Duke University School of Medicine, Durham, North Carolina, USA

## Abstract

**Background:**

Given the complexity of surgical care, perioperative patients are at high risk of opioid-related adverse drug events. Existing methods of detection, such as trigger tools and manual chart review, are time-intensive which makes sustainability challenging. Using strategic rule design, computerized surveillance may be an efficient, pharmacist-driven model for event detection that leverages existing staff resources.

**Methods:**

Computerized adverse drug event surveillance uses a logic-based rules engine to identify potential adverse drug events or evolving unsafe clinical conditions. We extended an inpatient rule (administration of naloxone) to detect opioid-related oversedation and respiratory depression to perioperative care at a large academic medical center. Our primary endpoint was the adverse drug event rate. For all patients with a naloxone alert, manual chart review was performed by a perioperative clinical pharmacist to assess patient harm. In patients with confirmed oversedation, other patient safety event databases were queried to determine if they could detect duplicate, prior, or subsequent opioid-related events.

**Results:**

We identified 419 cases of perioperative naloxone administration. Of these, 101 were given postoperatively and 69 were confirmed as adverse drug events after chart review yielding a rate of 1.89 adverse drug events/1000 surgical encounters across both the inpatient and ambulatory settings. Our ability to detect inpatient opioid adverse drug events increased 22.7% by expanding surveillance into perioperative care. Analysis of historical surveillance data as well as a voluntary reporting database revealed that 11 of our perioperative patients had prior or subsequent harmful oversedation. Nine of these cases received intraoperative naloxone, and 2 had received naloxone in the post-anesthesia care unit. Pharmacist effort was approximately 3 hours per week to evaluate naloxone alerts and confirm adverse drug events.

**Conclusion:**

A small investment of resources into a pharmacist-driven surveillance model gave great gains in organizational adverse drug event detection. The patients who experienced multiple events are particularly relevant to future studies seeking risk factors for opioid induced respiratory depression. Computerized surveillance is an efficient, impactful, and sustainable model for ongoing capture and analysis of these rare, but potentially serious events.

## Background

Perioperative care exists in a unique, highly complex environment comprised of preoperative screening, same day surgery, preoperative holding areas, operating rooms, and post-anesthesia care units (PACUs). Patient care is delivered by multidisciplinary teams, involves high cost, and utilizes sophisticated technologies that may have interoperability constraints [1,2]. The combination of these characteristics in such a fragmented environment creates high risk for medication-related harm [3]. Adverse drug events (ADEs) are defined as any injury resulting from medical interventions related to a drug, and they have been implicated in both increasing costs and length of stay within the surgical population [4]. It has been shown that inpatient clinical pharmacists' presence on care units can have a direct impact on increasing ADE discovery in critical care and pediatric settings [5,6]. Since much of the harm to surgical patients has been attributed to the lack of comprehensive oversight of high risk medications, the United States Pharmacopeia has recommended, and Duke has implemented, allocation of a dedicated pharmacist to the post-anesthesia care unit in order to oversee the distribution of medications [3].

Many models have been deployed to detect instances of medication-related harm in the surgical population. One study focused on opioid oversedation events found by retrospective chart review. They implemented successful strategies to reduce the incidence of these ADEs, but found it difficult to sustain improvements due to the lack of dedicated resources [7]. Another study used a structured survey tool of multiple open-ended questions administered to anesthesiologists. Trained examiners interviewed anesthesiologists in the PACU to elicit capture of non-routine events that may have caused patient harm. They concluded this methodology can detect a higher incidence of patient injury than chart review alone, and may be an effective strategy to complement voluntary reporting in anesthesia quality improvement (QI) [8]. Trigger tool methodology is designed for retrospective review of a random sample of patient charts using a list of "triggers," or rule sets. This targeted chart review streamlines effort by prompting the reviewer to seek specific indicators of care-induced injury. The Institute for Healthcare Improvement (IHI) developed a 28-trigger tool for surgical patients and described its use across 11 hospitals [9]. This tool has medication specific triggers, one involving the use of naloxone, and the results revealed that of 138 adverse events (AEs) identified in 125 patient records, 6 were opioid related. The authors concluded that the IHI surgical trigger tool is a useful methodology for detecting AEs in surgical patients and may serve as the basis for estimating AE rates that can be used to determine the impact of surgically-focused interventions.

Similarly, computerized ADE surveillance expands upon the trigger tool concept by using a computer program to query a hospital's clinical information system for a combination of characteristics that may indicate the occurrence of an ADE. Since a computer provides the first pass, manual chart review need only be performed for the potential events, thereby saving significant time over the traditional trigger tool method. Computerized surveillance technology has been implemented for both inpatient and ambulatory ADE detection and has shown promising results [10-12]. Although one study has used this methodology in the surgical inpatient population, we are unaware of any effort to deploy computerized surveillance across the perioperative continuum of care [13].

## Methods

### Study design

We performed a retrospective, cross-sectional study that included all surgical visits from 10/15/2007–10/15/2008 at Duke University Hospital (DUH). DUH perioperative care includes an inpatient/ambulatory center that contains 31 operating rooms (ORs), a preoperative and post-anesthesia care unit (PACU), an eye center with 6 ORs, and an ambulatory surgery center with 9 ORs. For the purposes of this study, we defined a surgical encounter as a single surgical case in an OR that may include one or more associated procedures. Although opioid medications are administered in several perioperative settings, the focus of our evaluation of patient harm was on postoperative oversedation since it is where clinical documentation is most complete. This study was approved by the Duke University Health System Institutional Review Board.

### Data collection

The computerized ADE surveillance (ADE-S) system is an internally developed application that evaluates inpatient medication, laboratory, and patient demographic information against a set of clinical rules to alert for potential ADEs or evolving unsafe conditions. Technical details of the system have been discussed in depth in prior publications [14,15]. Rules indicating situations requiring further evaluation include alerts for toxic serum drug levels, nephrotoxic medications and rising serum creatinine levels, or the administration of antidotes such as naloxone. Nearly 130 rules have been deployed since the system's inception (12/1/2004) to detect inpatient events. ADE-S delivers an electronic, daily report that details all triggers fired, which are then evaluated by clinical pharmacists for causality according to the method of Naranjo, and severity using the published Duke University Health System 7-point scale to ensure they are true instances of harm [16-18].

**Figure 1:**
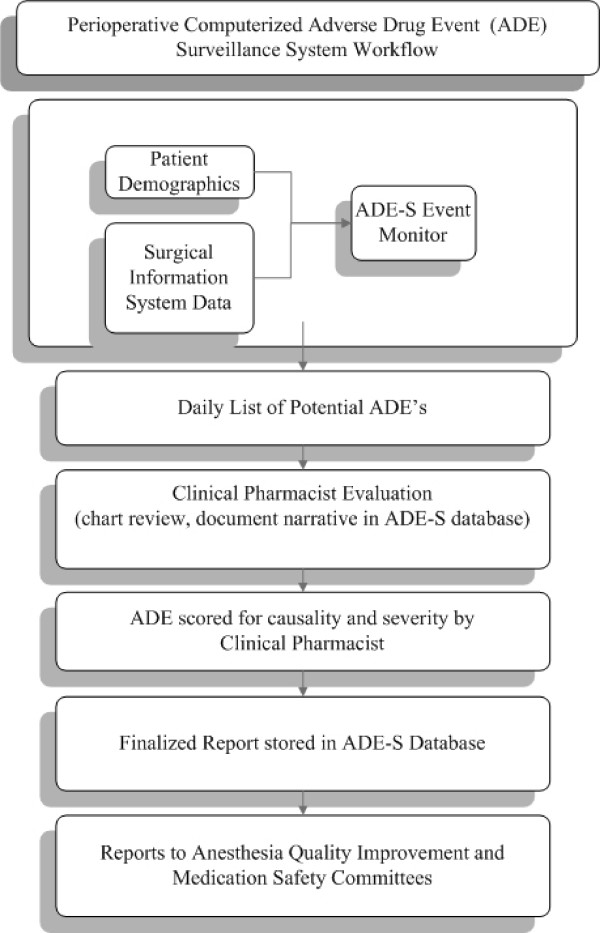
**Perioperative adverse drug event surveillance workflow**. Patient demographic information and surgical information system extracts are processed by the computerized surveillance rules engine. When trigger logic is met, results are populated in the adverse drug event surveillance (ADE-S) database. Patient charts and electronic medical record information are reviewed daily by a designated perioperative clinical pharmacist who then documents the details of the ADE in a clinical narrative within the ADE-S database. These completed evaluations are reviewed and scored by another clinical pharmacist for both causality and severity. Reports are generated for clinically-relevant analysis and quality improvement purposes.

On 10/15/2007, we added a rule that fires an alert upon administration of naloxone within a perioperative location. This information is found within the DUH surgical information system and is manually entered into an electronic anesthesia information system (Innovian [Drager, Telford, PA]) by anesthesia care providers and PACU nurses. Evaluation of the alerts within the ADE-S database (Figure 1) was incorporated to the daily workflow of an existing full time clinical pharmacist, who kept a daily log of time spent on the project. We defined an adverse event as 'an injury resulting from medical intervention related to a drug' [19]. The clinical pharmacist was trained to evaluate opioid-related oversedation events by the original developers of surveillance at DUH. This evaluation included an assessment of dosing, clinical monitoring of the patient, objective evidence of oversedation or respiratory depression, contributing medications, response to reversal with naloxone, assessment of alternative causes and risk factors, and the clinical outcome of the patient. We did not examine cases of the intraoperative use of naloxone in patients slow to emerge from anesthesia since usage is based on the clinical judgment of the anesthesiologist and the rationale was generally poorly documented in the anesthesia information system.

### Data analysis

Our primary endpoint is the ADE rate calculated as the number of ADE's per 1000 surgical encounters. The positive predictive value (PPV) of the naloxone alert was defined as the percentage of trigger alerts that resulted in an ADE. This is an established performance metric for the trigger [16]. As in most surveillance studies, sensitivity was not calculated as it was not feasible to perform the large number of chart reviews required to determine if there were oversedation events in patients who did not have alerts [16]. We performed a sub-analysis on inpatient and ambulatory surgical events where the respective rates were directly compared.

For those patients that were either a) administered intraoperative naloxone or b) had an ADE in PACU, we cross-referenced the surveillance (12/1/2006–11/5/2008) and voluntary incident reporting (4/1/2002–11/5/2008) databases to seek prior or subsequent instances of opioid-related harm. The voluntary reporting system permits website entry of any safety incident perceived by DUH staff.

## Results

**Figure 2:**
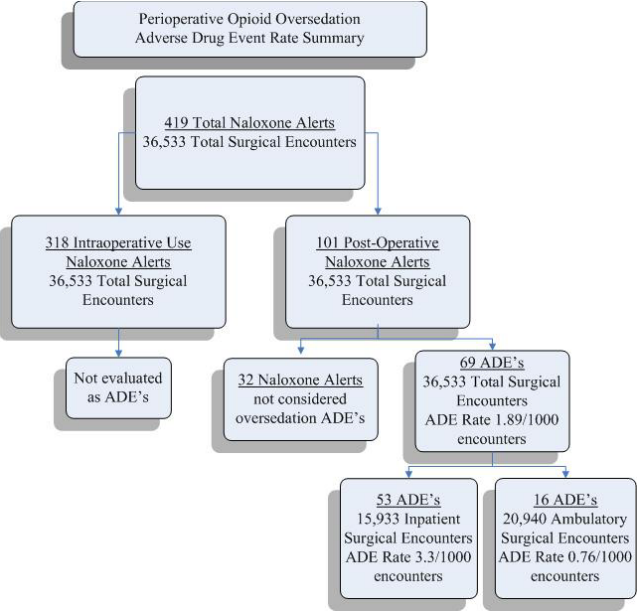
**Opioid-related ADE rates in perioperative care**. 419 Naloxone alerts in 36,533 surgical encounters are either due to intraoperative naloxone (318) or postoperative (101) post anesthesia care unit (PACU) usage. Of the 101 PACU cases, 69 were determined to be ADE's. ADE rates are further subdivided by inpatient and ambulatory subgroups.

From 10/15/2007–10/15/2008, 419 cases of naloxone administration across 36,533 DUH surgical encounters (15,993 inpatient and 20,940 ambulatory) were detected by the perioperative naloxone rule (Figure 2). Intraoperative naloxone administration accounted for 318 alerts which were not evaluated further. The remaining 101 alerts were indicative of naloxone administration in the post-operative period. These patient populations were mutually exclusive. Upon chart review of the post operative naloxone administration alerts, 69 were found to be ADEs due to perioperative opioid usage. The most common agents administered intraoperatively were fentanyl and hydromorphone. The overall PPV of the alert in the postoperative patients was 68.3% (69/101). Pharmacist effort was 3 hours/week based on a log maintained by the pharmacist.

The overall perioperative ADE rate was 1.89 ADEs/1000 surgical encounters (69/36,533). Fifty-three of the 69 ADEs occurred in inpatients, giving a rate of 3.3 ADEs/1000 inpatient surgical encounters (53/15,933). During this time, the overall inpatient DUH naloxone computerized surveillance rule detected 6.8 ADEs/1000 opioid-exposed patient encounters. Expanding detection into perioperative care therefore increased the number of discovered inpatient ADEs by 22.7%. Sixteen of the 69 postoperative ADEs occurred during ambulatory surgical procedures giving a rate of 0.76 ADEs/1000 ambulatory surgical encounters (16/20,940). Thirteen of the 16 ambulatory surgery patients who experienced ADEs postoperatively were subsequently discharged after the ADE resolved. Three ambulatory surgery patients were subsequently admitted due to surgical factors unrelated to the opioid ADE.

Of the 32 alerts for postoperative naloxone use that were not deemed as ADEs, 5 did indeed indicate patient harm but the ill effects were not sufficiently associated with an opioid to be considered an ADE in this study. Of the remaining 27 alerts, 3 cases had insufficient objective evidence of patient harm in the electronic health record, 13 were naloxone infusions for pruritus, and 11 were low-dose naloxone infusions for other clinical indications. Although some of these events may have been considered ADEs by a broader definition of causality, none were considered opioid-induced oversedation or respiratory depression.

For all patients receiving a naloxone trigger alert, we searched the ADE surveillance and voluntary reporting databases for either historical or subsequent opioid ADE history. This identified 11 unique cases of repeated harmful oversedation/respiratory depression in patients who received naloxone identified by the new surveillance rule (Table 1). Only one of these cases, however, was detected by voluntary reporting. Of all 11 subsequent opioid-related events, 6 occurred during the same hospital admission, 3 events occurred in a prior admission, and two occurred in a subsequent admission. Interestingly, the patient population that received intraoperative naloxone had a higher number of prior and subsequent events across these patient safety databases.

**Table 1:** Repeat Opioid-related ADEs in patients identified by a perioperative naloxone trigger

**ADE by Perioperative area**	**Same admission, Subsequent event**	**Prior Admission**	**Subsequent Admission**	**Total Repeat Events**
Intra-operative Naloxone	5	2	2	9

Post-Operative Naloxone	1	1 [a]	0	2

Total	6	3	2	11

[a] found by voluntary reporting system

## Discussion

Multiple methodologies have been used to quantify adverse drug events in the perioperative period including voluntary reporting systems, manual chart review, and trigger tools. These methods are often retrospective, resource intensive, and require highly-trained reviewers. A multi-center perioperative safety initiative that used the IHI surgical trigger tool illustrated that narcotic-related harm constituted 6 of the 138 adverse events identified from 854 patient record reviews (0.7/100 patient records) [9]. Another retrospective study that identified potential harm based on naloxone use and manual chart review reported a baseline oversedation rate of approximately 2 ADE's/1000 postoperative surgical patient discharges and required 20 hrs/week of clinical pharmacist effort [7]. Using targeted computerized detection methodology, we identified 3.3 ADEs/1000 surgical inpatient encounters with 3 hours/week effort. An inpatient study that used automated dispensing system charges for naloxone combined with targeted chart review demonstrated a PPV of 87% for this type of alert [20]. Our reported PPV was 68.3% in the postoperative population based on naloxone administration rather than dispensing. The difference in PPV may be attributable to a broader definition of an ADE compared to our focus only on oversedation/respiratory depression. In our review of the literature, we could not find any studies where the rate of opioid-related ambulatory ADEs was measured. Our ambulatory rate of 0.76 ADE's/1000 encounters is far less than the inpatient rate of 3.5 ADE's/1000 encounters, which may be reflective of the differences in the types of procedures performed, patients' comorbid conditions, acuity of presentation or other unidentified factors. We have expanded computerized ADE detection to reliably detect opioid-related oversedation in both the inpatient and ambulatory perioperative environment.

Sustainability of perioperative event detection is attributable to the incorporation of event evaluations into routine clinical pharmacist workflow. By expanding our methodology to the entire perioperative patient population, we were able to improve the overall detection of adverse events and compare surgical inpatients to overall inpatients. Our effort to cross reference other sources of ADE data supports the concept of using a broad approach to improve capture of events as part of an overall quality improvement strategy [21]. This study has also identified a subpopulation of patients that are of particular clinical interest – those that experience repeat incidences of oversedation. This subset of patients who experience repeated harmful events is important because it reveals that there may be underlying factors predisposing these patients to harm that have yet to be identified, and may require a more in-depth risk factor analysis. We are considering adding an alert to the patient's electronic health record that would signal if that patient has had a history of opiate-related ADEs and extra caution is necessary in drug dosage and monitoring. This may help prevent future ADEs.

There are several limitations to this study. In order for the computerized surveillance rule engine to detect naloxone administration, it must have been manually entered into the DUH anesthesia information system. Therefore, there is a remote chance that naloxone administration was not documented and a small number of alerts may have been missed. The ability to replicate the technical development of sophisticated computerized adverse drug event surveillance may not be readily transferable to other health systems, however vended systems are currently available. Additionally, we have chosen to focus only on a rare yet serious ADE due to opioids, although the trigger alert does potentially identify other types of ADEs. Patients most commonly received fentanyl or hydromorphone, but also received several other non-opioid medications with sedative effects. We did not analyze other potential ADE categories, such as the 13 trigger alerts due to naloxone infusions for pruritus or the 11 alerts for low-dose naloxone infusions to accompany other indications (e.g. patient controlled analgesia or epidurals). Finally, we did not evaluate the cases of intraoperative naloxone use more closely due to the lack documentation of the rationale for the administration of naloxone in the anesthesia information system.

Future work is needed to explore contributing factors for the oversedation cases both postoperatively as well as in the subpopulation of patients who received intraoperative naloxone. Since the intraoperative patients experienced a higher number of subsequent events, they are of particular interest. This may include the development of a predictive risk model or pharmacogenomic screening that could be used to spur intervention strategies that prevent events or at least their repetition [22]. As we implement quality improvement initiatives, such as special signaling for patients with a known history of oversedation, improved hand-off communications, increased monitoring for anyone experiencing an ADE postoperatively, and use of adjunctive opioid sparing non-sedating medications, we can use the computerized surveillance ADE rate as a quantitative measure to track longitudinal improvements. It is our hope that as electronic health records become mainstream that adverse drug event detection will evolve to creative point of care alerting and models will shift from detection toward mitigation of patient harm.

## Conclusion

ADE detection, evaluation, and analysis require considerable time, highly trained reviewers, and can be difficult to sustain long term. Success in this area depends on a reliable detection methodology, efficient incorporation into staff workflow, and ongoing measurement to differentiate short term benefits from sustained improvements. Our academic medical center has effectively leveraged an existing inpatient computerized ADE surveillance system and a dedicated perioperative clinical pharmacist to detect and evaluate perioperative adverse events. By expanding ADE detection to the immediate perioperative period using this pharmacist-driven model and focusing on the rare but serious event of opioid-related oversedation/respiratory depression, we have demonstrated a quantitative and sustainable model for improved ADE detection in surgical patients. We have further identified that intraoperative use of naloxone, and patients who experience repeat adverse events are areas in need of further investigation as we strive to improve the safety profile of our health system.

## Competing interests

The authors declare that they have no competing interests.

## Authors' contributions

JAE participated in the design of the study, data compilation and analysis, and drafted the manuscript. AW and KGG participated in data collection and revision of the manuscript. ASH, HC and JF participated in the design of the study, revision and approval of the manuscript. MMH performed data analysis and composed a critical revision of the manuscript for final submission.
